# Diffuse Ectopic Deciduosis Imitating Peritoneal Carcinomatosis with Acute Abdomen Presentation: A Case Report and Literature Review

**DOI:** 10.1155/2020/8847082

**Published:** 2020-09-25

**Authors:** Pavel Sorokin, Andrei Nikiforchin, Aleksandr Panin, Aleksandr Zhukov, Vadim Gushchin, Mark Kurtser

**Affiliations:** ^1^Department of Surgical Oncology, Lapino Clinical Hospital, 111 1st Uspenskoe Shosse, Lapino, Moscow Region, 143081, Russia; ^2^Department of Surgical Oncology, The Institute for Cancer Care, Mercy Medical Center, 227 Saint Paul Place, Weinberg Building, 4th Floor, Baltimore, Maryland, USA 21202; ^3^Department of General Surgery, Lapino Clinical Hospital, 111 1st Uspenskoe Shosse, Lapino, Moscow Region, 143081, Russia; ^4^Department of Pathology, Lapino Clinical Hospital, 111 1st Uspenskoe Shosse, Lapino, Moscow Region, 143081, Russia; ^5^Department of Obstetrics and Gynecology, Lapino Clinical Hospital, 111 1st Uspenskoe Shosse, Lapino, Moscow Region, 143081, Russia

## Abstract

During pregnancy, decidual tissue can occur beyond the endometrium, predominantly on the surface of the uterus, fallopian tubes, and ovaries. This condition, called ectopic deciduosis, generally is not accompanied by any symptoms and complications, does not require treatment, and resolves completely soon after labor. However, rarely it can present with acute abdomen syndrome or imitate peritoneal malignancy and, thus, cause diagnostic difficulties and unnecessary interventions. Here, we report a challenging case of a pregnant woman admitted with acute peritonitis caused by ectopic deciduosis that mimicked peritoneal carcinomatosis. This uncommon manifestation of deciduosis hindered correct diagnosis and led to excessive surgery. While the management of the patient presented is regrettable, the case highlights the natural history of deciduosis, and therefore, important lessons could be learned from it.

## 1. Introduction

Ectopic deciduosis refers to an abnormal occurrence of decidual tissue (decidua) outside the uterus [1, 2]. While its pathogenesis is not fully elucidated, it is thought to originate from subserous stromal cells as a result of progesterone stimulation [1–3]. The ectopic decidua appears on the surface of the female reproductive organs and peritoneum; however, on rare occasions, it can be found in the lymph nodes, lungs, kidneys, and skin [4–8].

Deciduosis is a benign condition that typically does not cause any symptoms and resolves spontaneously 4-6 weeks after labor [9, 10]. However, the ectopic decidua involving the appendiceal wall often leads to appendicitis and presents with signs of acute abdomen [11–13]. Diffuse omental and peritoneal involvement is another confusing manifestation that may mimic peritoneal carcinomatosis and mislead physicians [3, 10, 14]. A combination of both urgent surgical and oncology-like presentations of ectopic deciduosis may be even more diagnostically challenging making physicians prone to hasty clinical decisions. We report a rare case of a pregnant woman with this uncommon and complex manifestation of diffuse ectopic deciduosis that led to excessive surgery and undesirable outcome.

## 2. Case Presentation

A 36-year-old Caucasian woman (gravida 2, para 1) presented to the emergency room at 32-week gestation with acute lower abdominal pain, mild fever, and nausea. Before this admission, her pregnancy was uneventful and carefully screened. The patient had a history of myopia and laparoscopic removal of endometrioid ovarian cysts 3 years before. She never smoked and denied any cancer history. At presentation, she had a temperature of 37.8°C (100.1°F), her pulse rate was 105/min, and her blood pressure was 104/66 mmHg. Her respiratory rate was 21/min, and her oxygen saturation was 96% on room air. Physical examination revealed a 32-week gravid uterus and guarding with rebound tenderness in the right lower quadrant (RLQ) of the abdomen; bowel sounds were decreased. Laboratory tests showed a leukocyte count of 17,000/mm^3^ with 80% neutrophils. The levels of C-reactive protein (CRP) and interleukin 6 (IL-6) were elevated to 47 mg/L and 113 pg/mL, respectively. The performed abdominal and pelvic ultrasound (US) did not reveal an enlarged appendix or adnexa but showed slightly dilated bowel loops and a moderate amount of fluid in the RLQ and rectouterine pouch. The fetal heart rate was 160 beats/min. Due to suspected acute appendicitis, the patient underwent surgery.

**Figure 1:**
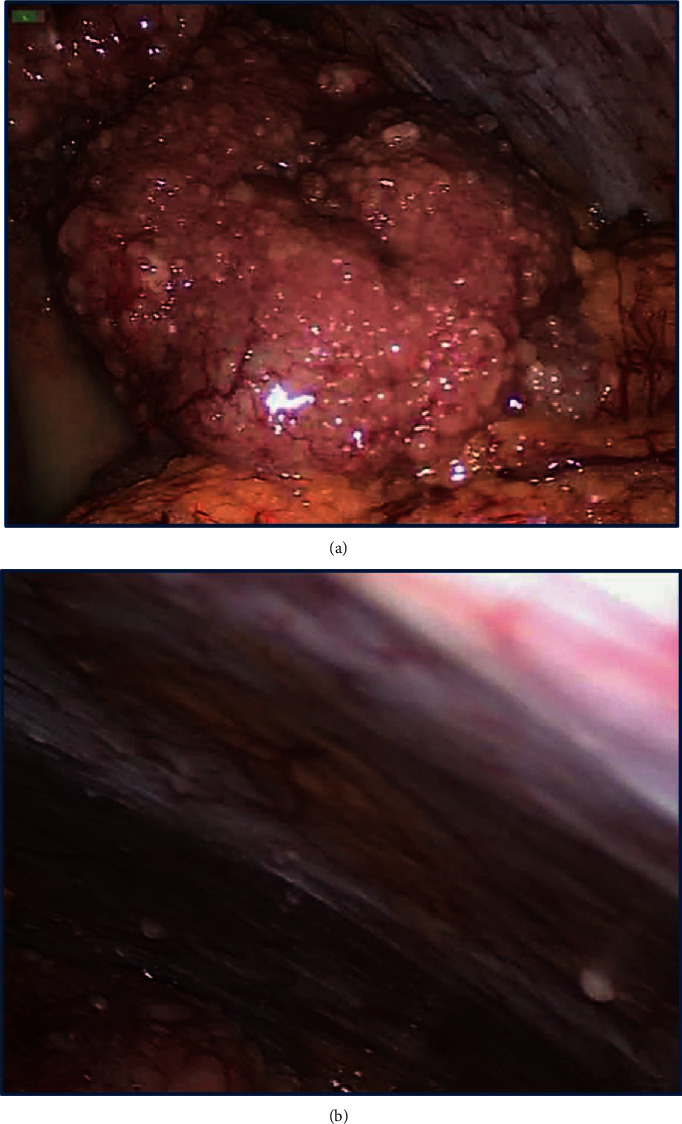
(a) A diagnostic laparoscopy demonstrates numerous ectopic decidual nodules covering the small bowel and imitating peritoneal carcinomatosis. There is a moderate amount of purulent-appearing fluid in the pelvis. (b) A diagnostic laparoscopy shows 3-5 mm nodules of ectopic deciduosis on the abdomen wall peritoneum.

The performed diagnostic laparoscopy revealed numerous 3-5 mm yellow peritoneal nodules on the small bowel and cecum serosa (Figure 1(a)). Similar lesions covered the surface of the uterus, enlarged ovaries and fallopian tubes, and partially the peritoneum of the abdominal wall and pelvis (Figure 1(b)). The subdiaphragmatic peritoneum, as well as the liver capsule, gallbladder, stomach, spleen, and appendix, was spared. Macroscopically, the lesions resembled peritoneal carcinomatosis, and the peritoneal cancer index (PCI) was 21 [15]. Purulent-appearing fluid was seen in the RLQ and pelvis and between inflamed and dilated bowel loops (Figure 1(a)). Peritoneal nodule biopsy was performed; however, the frozen sections were inconclusive, suggesting a neoplasm composed of spindle cells with unknown malignant potential. During the revision, the patient blood pressure decreased to 88/57 mmHg and she was started on fluid resuscitation with intravenous boluses. The patient was discussed intraoperatively with the obstetrician and surgical oncologist. Considering the severity of the patient condition (peritonitis, hypotension), the absence of a visible source of peritonitis (intact appendix, uninflamed adnexa), and the lack of sufficient visibility due to the gravid uterus, we decided to proceed with laparotomy and cesarean section. This decision was dictated by the risk of compromising the fetus and the necessity of a thorough revision of the abdomen to identify the origins of peritonitis and revealed lesions. After cesarean delivery, no gastrointestinal or pelvic source of peritonitis was found; therefore, the necrosis of peritoneal lesions was concluded to be its cause. Interpreting the peritoneal nodules as carcinomatosis and considering ovarian, tubal, and peritoneal cancer its most common origin in women, we performed bilateral salpingoophorectomy with omental and multiple peritoneal biopsies for further histopathology confirmation.

**Figure 2:**
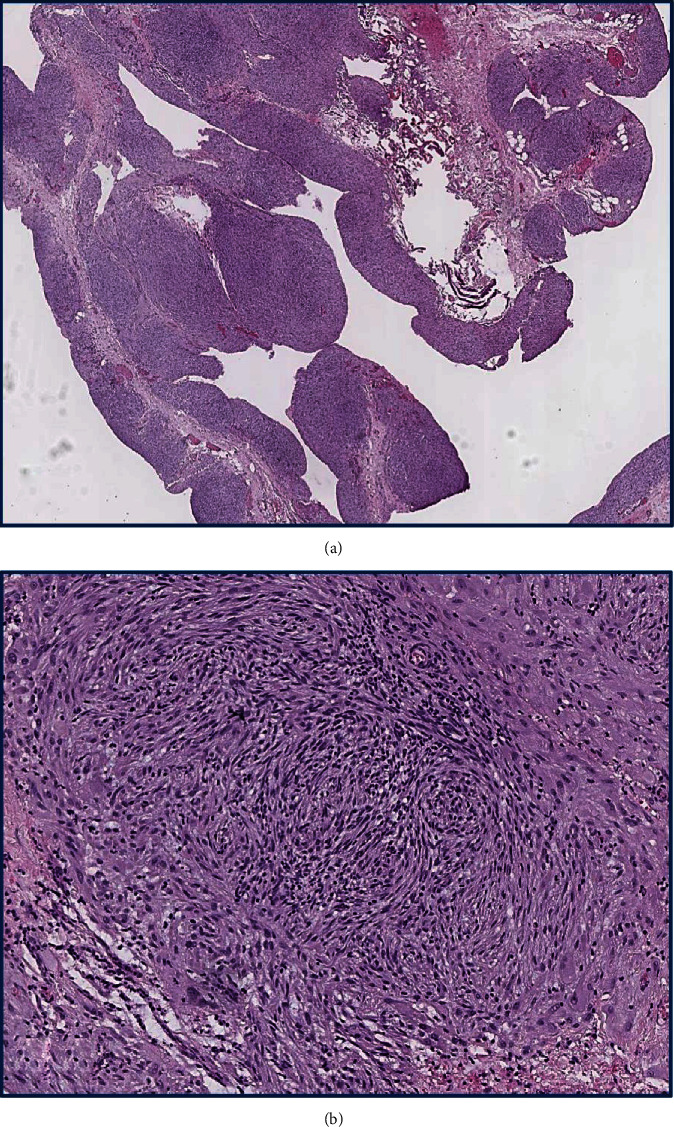
(a) Microscopic examination of the peritoneum sample shows several foci of atypical tissue growth (H&E, magnification ×50). (b) Microscopic examination of the peritoneal lesion demonstrates nodes and fields of large spindle and oval cells with uniform nuclei and a moderately developed cytoplasm, forming bundles and swirls (H&E, magnification ×400). H&E: hematoxylin and eosin.

**Figure 3:**
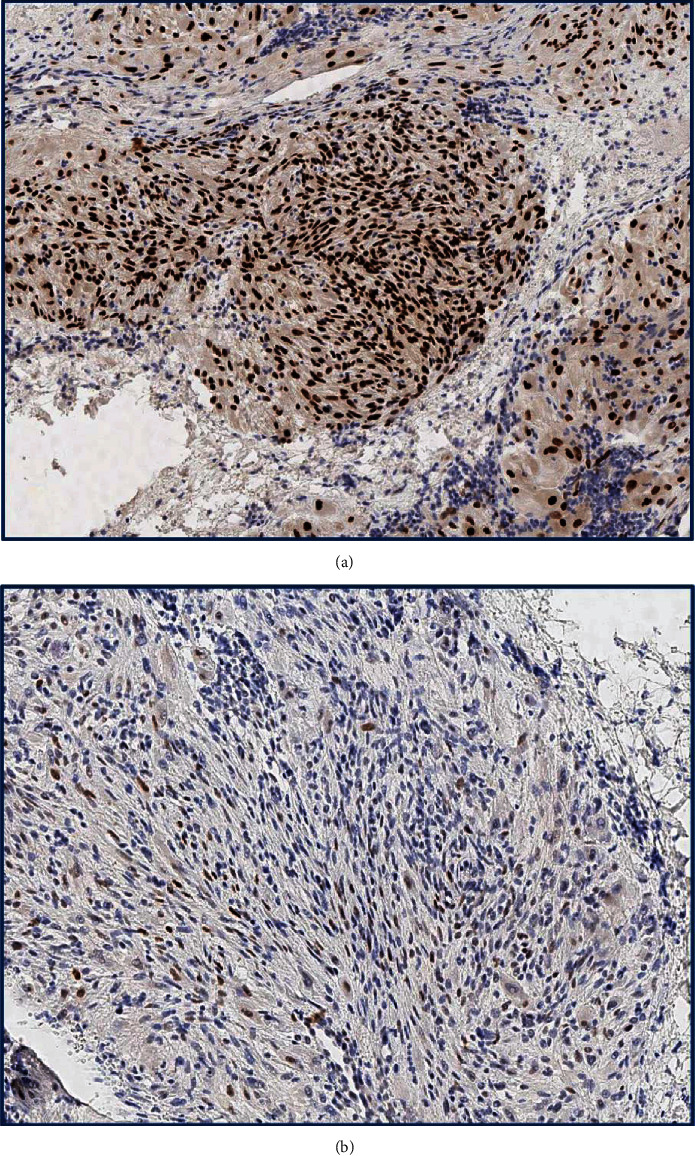
(a) The diffuse nuclear PR expression in decidually transformed cells (magnification ×400). (b) The mild nuclear ER expression in decidually transformed cells (magnification ×400). PR: progesterone receptors; ER: estrogen receptors.

**Figure 4:**
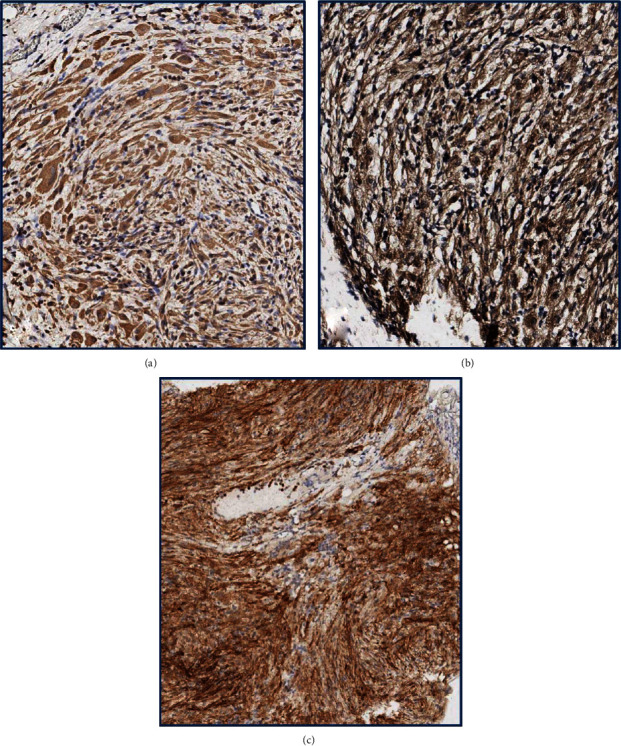
(a) The intensive diffuse cytoplasmic expression of vimentin in decidually transformed cells (magnification ×400). (b) The diffuse cytoplasmic desmin expression in decidually transformed cells (magnification ×400). (c) The intensive membrane and cytoplasm expression of CD10 in decidually transformed cells (magnification ×400). CD10: cluster of differentiation 10.

The postoperative evaluation of suppurative fluid did not show bacterial growth. The final pathology of sampled tissues did not reveal any signs of malignancy and reported submesothelial fields of spindle and oval cells, forming bundles and swirls suspicious of decidual transformation (Figures 2(a) and 2(b)). The immunohistochemical (IHC) assay demonstrated coexpression of progesterone (PR) and estrogen receptors (ER) (Figures 3(a) and 3(b)), vimentin, desmin, and the cluster of differentiation 10 (CD10) (Figures 4(a)–4(c)), but there was no staining for calretinin, cytokeratin 5, smooth muscle actin (SMA), c-kit (CD117), human melanoma black 45 (HMB-45), and S-100 protein, which is consistent with deciduosis criteria.

**Figure 5:**
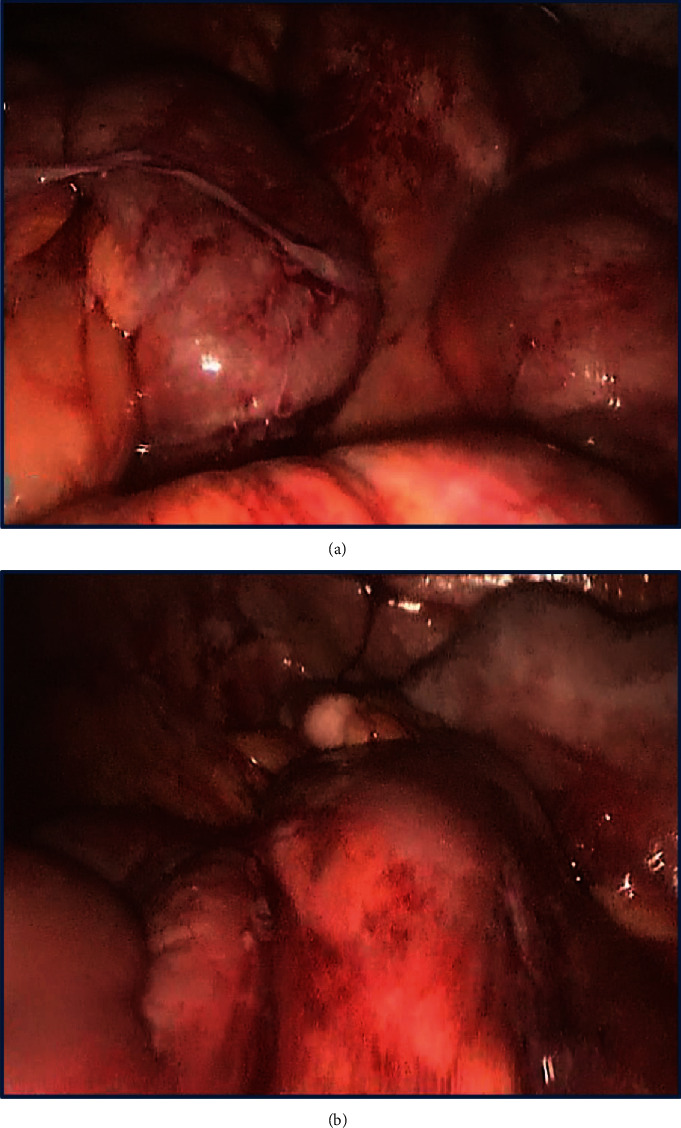
(a) A control diagnostic laparoscopy 6 weeks after cesarean delivery shows a full regression of ectopic deciduosis that covered small bowel loops. (b) A control diagnostic laparoscopy 6 weeks after cesarean delivery shows an entire regression of ectopic deciduosis that used to cover the terminal ileum and cecum completely.

The patient recovered uneventfully and was discharged on the 6^th^ postoperative day. After delivery, the 2100 g (4 lb 10 oz) male newborn was transferred to the neonatal intensive care unit and discharged one week after. The patient underwent a control diagnostic laparoscopy six weeks after surgery that showed the complete regression of peritoneal nodules (Figures 5(a) and 5(b)). Given the premature surgical menopause, the patient was offered hormone therapy with estrogen which she refused. At six months of follow-up, the patient and her child do not have any symptoms or concerns.

## 3. Discussion

This case demonstrates management challenges that physicians may face due to an uncommon presentation of ectopic deciduosis. As a rule, this obstetric condition does not cause any symptoms or laboratory abnormalities and resolves spontaneously 4-6 weeks after delivery [1, 9, 14]. However, if the appendix wall undergoes a decidual transformation, it can lead to appendicitis with typical clinical and laboratory characteristics [11–13]. Also, there are a number of reports describing other urgent manifestations caused by diffuse ectopic deciduosis including intraperitoneal hemorrhage, tuboovarian abscess, bowel obstruction, and dystocia [11, 16–20]. In our case, the symptoms and physical examination indicated acute abdomen syndrome, and elevated inflammatory markers (neutrophilic leukocytosis, CRP, and IL-6) corroborated the clinical impression. When presented as acute abdomen syndrome, ectopic deciduosis requires further thorough investigation to rule out other common causes of this surgical emergency [21].

Imaging can significantly contribute to the differential diagnosis of acute lower abdominal pain during pregnancy and identify its causes, more common than ectopic deciduosis. The abdominal and pelvic US should be an initial test since it is noninvasive, not associated with ionizing radiation exposure, and informative in acute appendicitis or gynecological emergencies [22, 23]. Magnetic resonance imaging (MRI) is also preferable as it avoids ionizing radiation and not inferior to computed tomography in the evaluation of acute nontraumatic abdominal pain during pregnancy [21, 24]. Unfortunately, ectopic deciduosis itself is usually unseen on imaging due to the insufficient size of nodules and indifferent tissue density. In the present case, ultrasonography did not reveal an enlarged appendix or adnexa but it visualized a moderate amount of fluid in the RLQ and rectouterine pouch. Based on physical examination and laboratory test results, we suspected acute appendicitis, the most common surgical emergency in pregnancy [25]. A diagnostic laparoscopy was chosen as the next minimally invasive and safe management step allowing both abdominal revision and curative surgery [21, 26].

The macroscopic appearance of the ectopic decidua is insidious as it lacks specific features and can be easily mistaken for a tumor. In general, it presents as small yellow to tan elastic, sometimes focally hemorrhagic, nodules or plaques localized on the surface of the uterus, fallopian tubes, ovaries, and pelvic peritoneum without any exudate [1, 2, 4, 9]. Diffuse involvement of the peritoneum and abdominal organs is rare and thus can be especially challenging for diagnosis because it imitates peritoneal carcinomatosis [3, 9, 10, 14]. In the present case, the laparoscopy suggested peritonitis along with numerous small yellow nodules covering the uterus, fallopian tubes, enlarged ovaries, peritoneum, loops of the small bowel, and cecum (Figures 1(a) and 1(b)). The severity of the patient condition (peritonitis, hypotension) required rapid decision-making, and right after intraoperative discussion with the obstetrician, cesarean section was performed to reduce the fetal risks. However, after delivery, no distinct source of peritonitis was identified, and the frozen section of the lesions suggested a spindle cell neoplasm with unknown malignant potential. The surgical oncologist interpreted the intraoperative findings as carcinomatosis from the ovarian primary tumor with decay that led to peritonitis (the postoperative assessment of suppurative fluid did not show bacterial growth). Although the incidence of ovarian, tubal, and peritoneal cancer during pregnancy is low, it remains the most common origin of peritoneal carcinomatosis in women, and since the appendix, stomach, colon, liver, and gallbladder were intact, all specialists agreed on this diagnosis [27, 28]. An extensive spread of lesions was interpreted as an advanced stage of the disease with PCI 21. The striking macroscopic resemblance of deciduosis with peritoneal carcinomatosis contributed to major departure from common sense and unnecessary further surgery. Analyzing the case retrospectively, the initial intervention should have been limited to appropriate drainage and peritoneal and omental biopsies which are sufficient for establishing a diagnosis when ovarian cancer is suspected [29]. Thus, in pregnant women with peritoneal lesions, ectopic deciduosis should be always taken into account since its gross anatomy can be mistaken for carcinomatosis and no intraoperative decisions should be made based on sole macroscopic appearance.

Generally, ectopic deciduosis is a self-limited condition that resolves completely in the early postpartum period and on its own not requiring any treatment [9]. However, surgical intervention may be needed in case of acute appendicitis or a tuboovarian abscess caused by the decidual transformation of corresponding tissues [11, 12]. Other rare complications including intraperitoneal hemorrhage and bowel obstruction may also require surgery when they do not respond to conservative measures [16, 18, 19]. Pregnancy management is another important component of care of these patients, and the obstetrician should be involved even though the vast majority of cases will end with at-term delivery [9, 10]. In the case of such a challenging manifestation of ectopic deciduosis such as peritonitis and peritoneal lesions, we also recommend a surgical oncologist consultation for comprehensive differential diagnosis and deliberate decision-making. Importantly, regardless of clinical presentation, if the surgery is performed, it should always include sufficient biopsy of the decidua for further histopathology evaluation.

A thorough pathological assessment of surgical specimens, which is the key in making a diagnosis of ectopic deciduosis, requires time and resources and cannot be performed intraoperatively with frozen sections. In general, microscopic evaluation of the decidua shows large cells with spindle, oval, or polygonal shape that form bundles or clusters in the submesothelial layer [1, 2, 10]. Decidual tissue is benign so it typically does not demonstrate increased mitotic activity, nuclear pleomorphism, necrosis, and vascular invasion [1, 2]. A broad IHC assay should be also performed to distinguish the ectopic decidua from some neoplasms that have similar macro- and microscopic appearance [2]. The expression of PR, ER, vimentin, desmin, and CD-10 showed to be specific for deciduosis, while calretinin and cytokeratin 5/6 positivity supports deciduoid malignant mesothelioma [2, 9, 14]. To rule out metastatic melanoma, IHC should demonstrate negativity for HMB-45 and S-100 protein stains, while the negativity for c-kit (CD117) excludes gastrointestinal stromal tumors [2, 14]. In the reported case, the final pathology revealed the fields of spindle and oval cells forming bundles and swirls (Figures 2(a) and 2(b)) that along with an expression of PR, ER (Figures 3(a) and 3(b)), vimentin, desmin, and CD10 (Figures 4(a)–4(c)) confirmed ectopic deciduosis. Sufficient biopsy and meticulous pathology evaluation with the IHC analysis are crucial for accurate diagnosis of ectopic deciduosis.

## 4. Conclusion

The presented case demonstrates diagnostic challenges caused by an uncommon manifestation of ectopic deciduosis that led to excessive surgery. This benign obstetric condition should be always kept in mind for differential diagnosis when the peritoneal spread of tumor-like lesions is found during pregnancy and the early postpartum period, and sufficient but not excessive biopsy of suspicious nodules is required for an accurate diagnosis. We believe that our clinical experience and its analysis will aid physicians in the appropriate management and reduce the risk of undesirable outcomes.

## Data Availability

The data used to support the findings of this study is restricted by the Ethical Committee of Lapino Clinical Hospital in order to protect patient privacy. Data is available upon request from the corresponding author for researchers who meet the criteria for access to confidential data.
